# PEDF Inhibits the Activation of NLRP3 Inflammasome in Hypoxia Cardiomyocytes through PEDF Receptor/Phospholipase A2

**DOI:** 10.3390/ijms17122064

**Published:** 2016-12-12

**Authors:** Zhongxin Zhou, Zhu Wang, Qiuhua Guan, Fan Qiu, Yufeng Li, Zhiwei Liu, Hao Zhang, Hongyan Dong, Zhongming Zhang

**Affiliations:** 1 Department of Thoracic Cardiovascular Surgery, Affiliated Hospital of Xuzhou Medical University, 99 Huaihai Road, Xuzhou 221006, China; zzzgqh@163.com (Z.Z.); wzxy1984@163.com (Z.W.); viovan@163.com (F.Q.); 18761437629@163.com (Y.L.); zhanghao@xzmc.edu.cn (H.Z.); 2 Research Center for Biochemistry and Molecular Biology and Provincial Key Laboratory of Brain Disease Bioinformation, Xuzhou Medical University, Xuzhou 221004, China; guanqiuhua2008@163.com; 3 Research Facility Center for Morphology, Xuzhou Medical University, 209 Tongshan Road, Xuzhou 221004, China; gavinliu@xzmc.edu.cn

**Keywords:** pigment epithelium-derived factor (PEDF), pigment epithelial-derived factor receptor (PEDFR), nucleotide-binding oligomerization domain-like receptor protein 3 (NLRP3) inflammasome, mitochondrial fission, dynamin-related peptide 1 (Drp1)

## Abstract

The nucleotide-binding oligomerization domain-like receptor protein 3 (NLRP3) inflammasome has been linked to sterile inflammation, which is involved in ischemic injury in myocardial cells. Pigment epithelium-derived factor (PEDF) is a multifunctional secreted glycoprotein with many biological activities, such as anti-inflammatory, antioxidant and anti-angiogenic properties. However, it is not known whether and how PEDF acts to regulate the activation of the NLRP3 inflammasome in cardiomyocytes. In the present study, we used the neonatal cardiomyocytes models of ischemia-like conditions to evaluate the mitochondrial fission and the activation of the NLRP3 inflammasome. We also determined the mechanism by which PEDF inhibits hypoxia-induced activation of the NLRP3 inflammasome. We found that PEDF decreased the activation of the NLRP3 inflammasome in neonatal cardiomyocytes through pigment epithelial-derived factor receptor/calcium-independent phospholipase A2 (PEDFR/iPLA2). Meanwhile, PEDF reduced Drp1-induced mitochondrial fission and mitochondrial fission-induced mitochondrial DNA (mtDNA), as well as mitochondrial reactive oxygen species (mtROS) release into cytosol through PEDFR/iPLA2. We also found that PEDF inhibited mitochondrial fission-induced NLRP3 inflammasome activation. Furthermore, previous research has found that endogenous cytosolic mtDNA and mtROS can serve as activators of NLRP3 inflammasome activity. Therefore, we hypothesized that PEDF can protect against hypoxia-induced activation of the NLRP3 inflammasome by inhibiting mitochondrial fission though PEDFR/iPLA2.

## 1. Introduction

Acute myocardial infarction (AMI) is a world-wide cardiovascular disease that leads to serious consequences for society [1,2]. During AMI, an intense inflammatory response occurs, which brings adverse outcomes [3]. The inflammatory process is essential for tissue healing, but may also lead to over-injury and poorly-adapted ventricular remodeling, resulting in impaired myocardial function and heart failure [4]. There is growing evidence that the innate immune system participates in regulating the myocardial response to tissue injury [5].

The nucleotide-binding oligomerization domain-like receptor protein 3 (NLRP3) inflammasome is a molecular platform activated upon signs of cellular “danger” to trigger innate immune defenses through the maturation of pro-inflammatory cytokines, such as interleukin (IL)-1β and IL-18 [6]. Once activated, NLRP3 will form a complex with its adaptor apoptosis-associated speck-like protein containing a caspase recruitment domain (ASC), to facilitate the autocatalytic activation of pro-caspase-1 and the formation of an active caspase-1 p10/20 tetramer [7]. The activated caspase-1 can induce a distinct form of programmed cell death called “pyroptosis”. In addition to inducing “proptosis”, the activated caspase-1 can also process pro-IL-1β and pro-IL-18 into their mature forms, which are important in cardiomyocyte apoptosis [7,8]. An ever-increasing number of studies links a direct pathophysiological role of NLRP3 inflammasome activation to ischemic cardiovascular diseases [9,10] and, thus, provides a new treatment regimen for cardiovascular diseases.

PEDF, a 50-kDa secreted glycoprotein, is a member of serine protease inhibitors superfamily [11]. It is expressed in multiple tissues and exerts diverse physiological activities, for instance blocking angiogenesis and tumor growth, anti-inflammatory and anti-oxidation [12,13,14]. Our previous studies have declared that PEDF inhibits left ventricular remodeling and improves cardiac function in rats with acute myocardial infarction, and PEDF protects against hypoxia-induced apoptosis and necroptosis via an anti-oxidative effect [15,16]. However, it is not known whether PEDF inhibits the activation of the NLRP3 inflammasome to protect against myocardial cell death in hypoxia cardiomyocytes.

A plurality of membrane receptors of PEDF has been identified in different cell types. The laminin receptor (LR) is a PEDF receptor, which induces endothelial cell (EC) apoptosis and inhibits EC migration, tube-like network formation in vitro and retinal angiogenesis ex vivo [17]. Current evidence demonstrates that the 80-kDa PEDFR is another receptor for PEDF in retinal epithelial cells [18]. PEDF binds to PEDFR on H9C2 cell membranes [19]. PEDFR indicates a high-affinity for PEDF. Interestingly, after PEDF binding, PEDFR presents a potent phospholipase A2 (PLA2) enzymatic activity and lipase activity [20]. Recently, our previous study indicated that PEDF attenuates hypoxia-induced apoptosis and necrosis in H9C2 cells by inhibiting p53 mitochondrial translocation via PEDFR [19]. However, it is not clear which receptor of PEDF plays a role in inhibiting the activation of the NLRP3 inflammasome.

In this study, we mainly used cultured neonatal cardiomyocytes to observe whether PEDF plays a protective role in hypoxia cardiomyocytes though inhibiting the activation of the NLRP3 inflammasome and explore the mechanism by which PEDF mediates this effect.

## 2. Results

### 2.1. PEDF Decreased the Expression of the NLRP3 Inflammasome and Mitochondrial Fission in the Rat Heart during AMI

**Figure 1:**
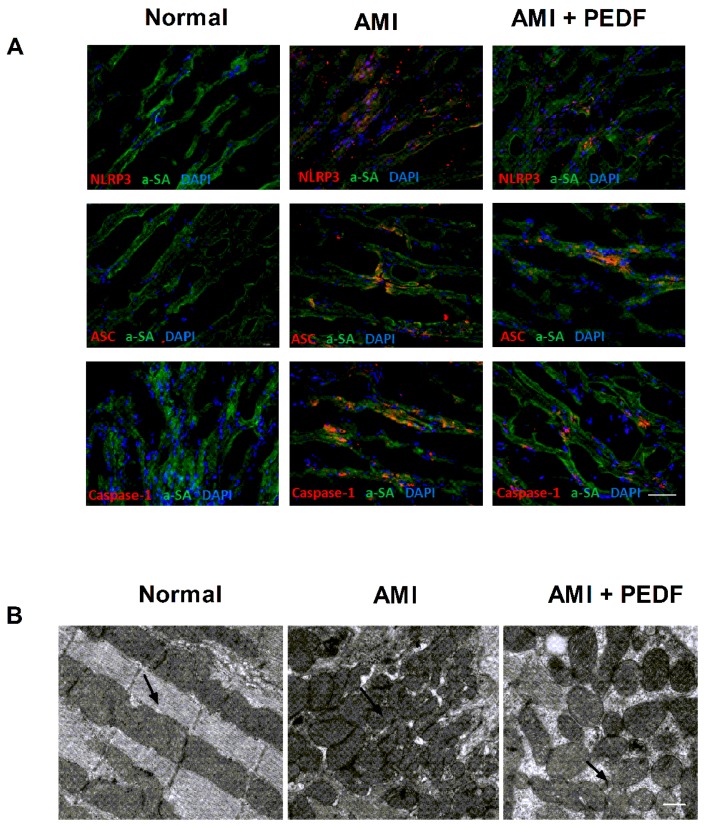
Formation of the inflammasome in the cardiomyocytes. (**A**) Immunofluorescence shows the overlap of a-sarcomeric (α-SA) staining (green) and NLRP3/ASC/caspase-1 (p20) (red) in the border zone, 7 day after acute myocardial infarction (AMI). Counterstaining with 4’,6-Diamidino-2-phenylindole, dihydrochloride (DAPI) (blue), original magnification 40× (scale bar = 20 μm); (**B**) electron micrograph of adult murine heart. The pictures are representative of three independent experiments. Normal, mitochondria of normal lengths (one or two mitochondria per sarcomere). AMI, representative electron micrograph of adult murine heart depicting fragmented mitochondria with disorganized cristae 7 days after AMI. PEDF, under the PEDF condition mitochondria, were able to undergo end-to-end fusion. Positive mitochondria indicated by black arrow, Magnification = 1000× (scale bar = 500 nm).

Immunofluorescence staining for the NLRP3 inflammasome appears as perinuclear cytoplasmic aggregates in cardiomyocytes in the infarct border zones, with virtually no staining in the hearts of sham-operated mice and highly expressed in the cytoplasm of monoclonal anti-actin a-sarcomeric (α-SA) staining cardiomyocytes bordering the infarct 7 days after AMI. PEDF treatment decreased NLRP3 inflammasome expression in the heart 7 days after AMI (Figure 1A). These results clearly indicate that PEDF remarkably decreases the expression of NLRP3 and caspase-1 (p20) in cardiomyocytes after AMI.

The effect of PEDF on mitochondrial morphology was also examined in heart 7 days after AMI. The results of the electron micrograph of adult murine hearts showed that in normal hearts, the mitochondria mainly displayed an organized and elongated morphology. However, mitochondria became numerous, smaller and spherical following AMI. After Recombinant PEDF-lentivirus (PEDF-LV) treatment, the fragmentation of the numerous smaller spherical mitochondria changed into elongated and interconnected mitochondria in the heat of AMI (Figure 1B). These results clearly indicated that the expression of the NLRP3 inflammasome and mitochondrial fission in the PEDF group were remarkably inhibited.

### 2.2. Hypoxia Induced the Activation of NLRP3 Inflammasome in Cultured Neonatal Cardiomyocytes

**Figure 2:**
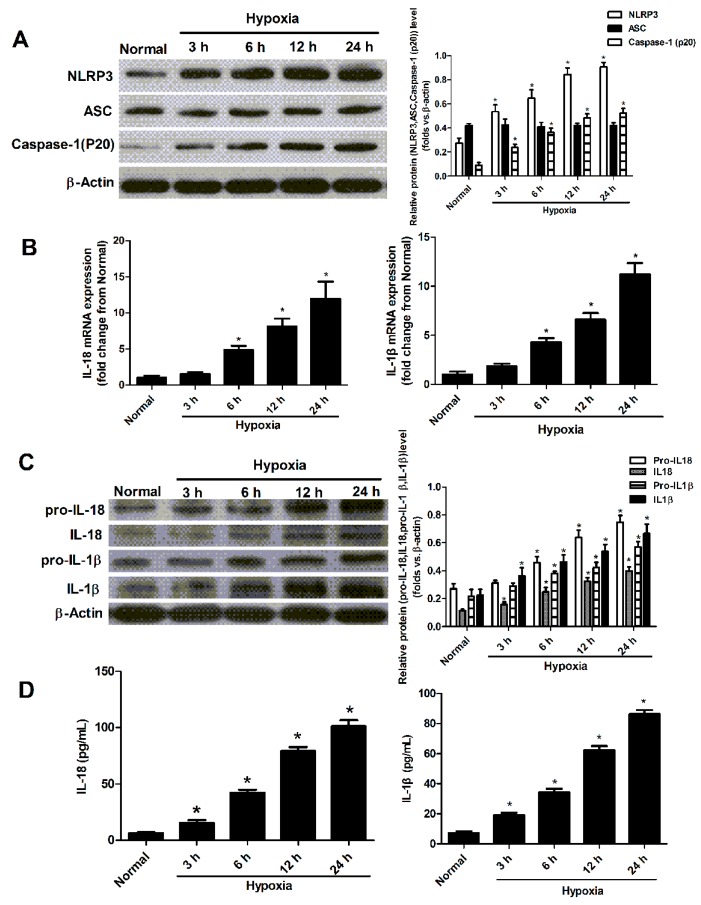
The activation of the NLRP3 inflammasome in neonatal cardiomyocytes. (**A**) Western blot analyzed the expression of NLRP3, ASC and caspase-1 at 3, 6, 12, 24 h after hypoxia (*n* = 4, * *p* < 0.05 vs. the normal group); (**B**) the mRNA expression of IL-1β, IL-18 was examined by real-time PCR (*n* = 4, * *p* < 0.05 vs. the normal group); (**C**) Western blot analyzed the expression of pro-IL18/1β and IL18/1β at 3, 6, 12 and 24 h after hypoxia (*n* = 4, * *p* < 0.05 vs. the normal group); (**D**) ELISA analyzed the protein levels of IL-1β and IL-18 in the cultured supernatants of neonatal cardiomyocytes. Data are presented as the means ± SD (*n* = 4, * *p* < 0.05 vs. the normal group). The significance among the normal and hypoxia groups within different stimulated times.

To determine whether ischemia-like conditions activate the NLRP3 inflammasome in cardiomyocytes, we evaluated the temporal expression of all NLRP3 inflammasome components in myocardial cells subjected to hypoxia. First, we measured the protein levels of NLRP3, ASC and caspase-1 (p20) after hypoxia in neonatal cardiomyocytes at different time point. The levels of the NLRP3 and caspase-1 (p20) proteins increased within 3 h of exposure to hypoxia and remained elevated for 24 h. The protein level of ASC remained unchanged in hypoxic conditions (Figure 2A). Then, we evaluated the temporal expression of interleukin (IL)18/1β mRNA and protein levels of pro-IL18/1b and IL18/1b in myocardial cells subjected to hypoxia. The mRNA levels of IL18 and IL-1β in myocardial cells were raised at 6 h after hypoxia stimulation (Figure 2B). The IL18/1β proteins increased within 3 h of exposure to hypoxia and remained elevated for 24 h. The pro-IL18/1β began to increase at 6 h under hypoxia condition (Figure 2C). The secreted levels of IL-1β and IL-18 in the cultured supernatant of neonatal cardiomyocytes also increased within 3 h and remained elevated for 24 h (Figure 2D).

### 2.3. PEDF Inhibited Hypoxia-Induced NLRP3 Inflammasome Activation via PEDF-R/iPLA2 in the Neonatal Cardiomyocytes

To assess whether PEDF inhibits hypoxia-induced NLRP3 inflammasome activation via PEDFR or laminin receptor (LR) in cardiomyocytes, the neonatal cardiomyocytes were treated with PEDF under hypoxic conditions, and RNA interference assays were used to silence PEDFR and LR. Inhibitors were used to block the activity of iPLA2 and lipase activity.

**Figure 3:**
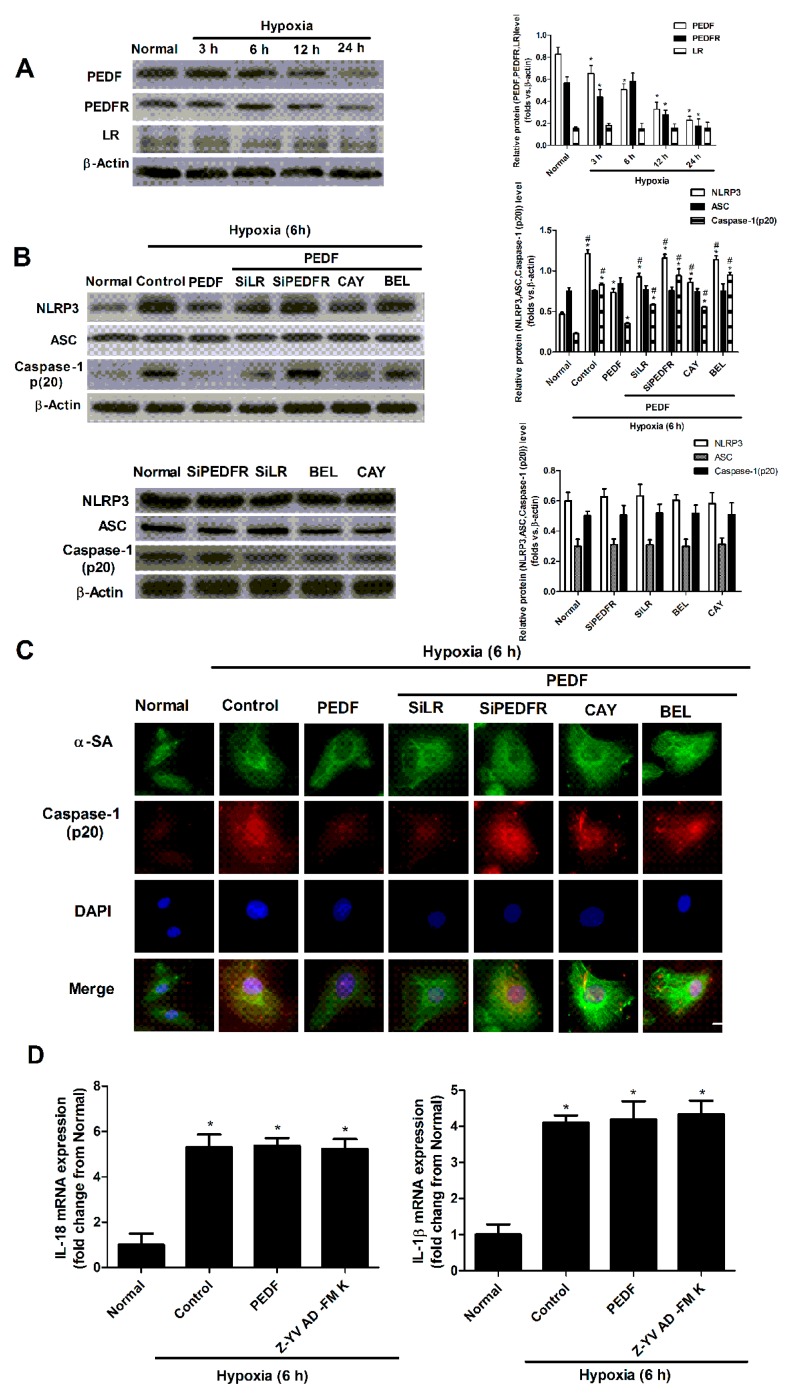

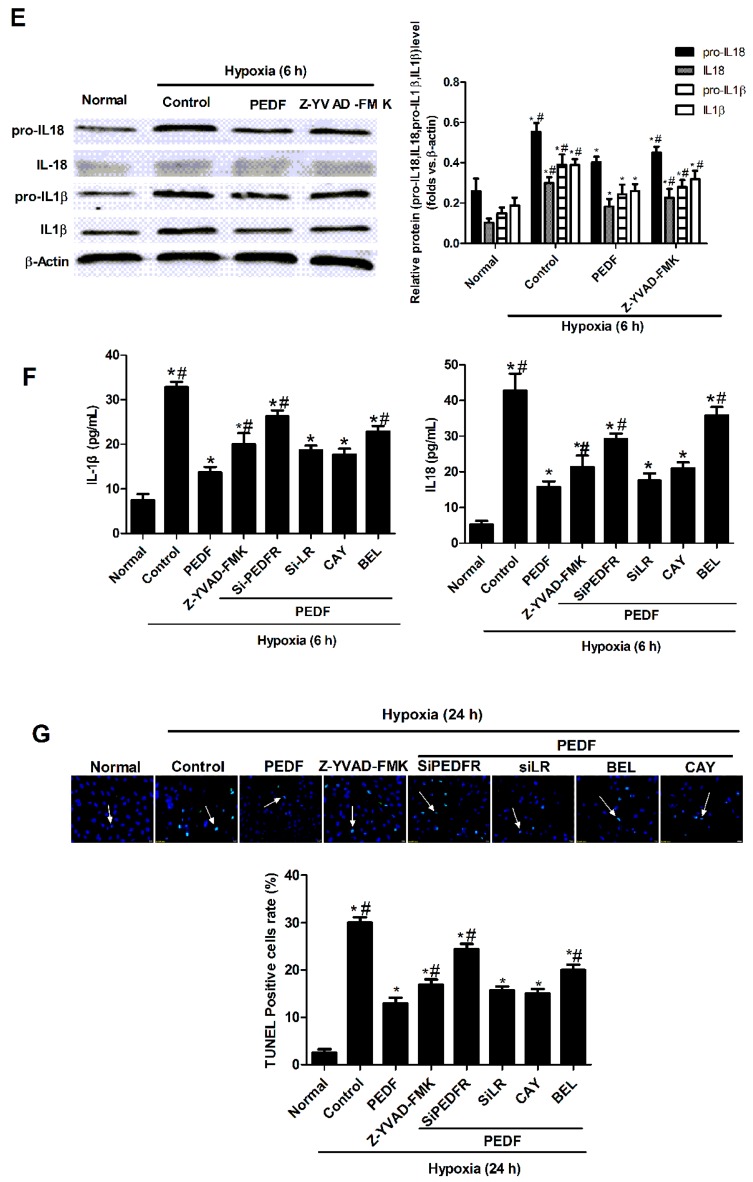
NLRP3 inflammasome activation in the neonatal cardiomyocytes and cell apoptosis. (**A**) Western blot analyzed the expression of PEDF, PEDFR and LR proteins at 3, 6, 12 and 24 h after hypoxia (*n* = 4, * *p* < 0.05 vs. the normal group); (**B**) Western blot illustrated the effect of PEDF (10 nM) treatment on the expression levels of NLRP3 inflammasome proteins, following hypoxia (6 h) in neonatal cardiomyocytes (*n* = 4; * *p* < 0.05 vs. the control group; # *p* < 0.05 vs. the PEDF group); (**C**) immunofluorescence analyzed caspase-1 (p20) in neonatal cardiomyocytes; scale bar: 20 µm; (**D**) IL-1β/18 mRNA expression was examined by real-time PCR; the results were expressed as the relative expression to β-actin and plotted as the ratio of the control group (*n* = 4. * *p* < 0.05 vs. the control group; # *p* < 0.05 vs. the PEDF group); (**E**) representative Western blot analyses of pro-IL18/1β and IL-1β/18 expression (*n* = 4. * *p* < 0.05 vs. the control group; # *p* < 0.05 vs. the PEDF group); (**F**) ELISA analyzed the protein levels of IL-1β and IL-18 after hypoxia (6 h) (*n* = 4. * *p* < 0.05 vs. the control group; # *p* < 0.05 vs. the PEDF group); (**G**) Terminal deoxynucleotidyl transferase-mediated dUTP nick-end-labeling (TUNEL) staining for cardiomyocyte apoptosis (green), DAPI for nuclear staining (blue); apoptosis cell indicated by white arrow; scale bar: 20 µm (*n* = 6. * *p* < 0.05 vs. the control group; # *p* < 0.05 vs. the PEDF group). Data are expressed as the mean ± SD.

Firstly, we examined the protein (PEDF, PEDFR and LR) expression levels in the neonatal cardiomyocytes after hypoxia (Figure 3A). The protein levels of PEDF in cells significantly decreased 3 h after hypoxia compared with the normal group and remained decreased during the observational periods. However, the protein level of PEDFR began to decrease at 6 h after hypoxia. The protein level of LR was very low and remained unchanged in hypoxic conditions (Figure 3A).

Western blotting and immunofluorescence showed that at 6 h after hypoxia, NLRP3 and caspase-1 (p20) proteins were increased significantly. PEDF reduced the hypoxia-induced expression of NLRP3 and caspase-1 (p20) at 6 h after hypoxia. PEDF’s effect was abolished by PEDF-R siRNA, but not the LR siRNA, which also was abolished by BEL (iPLA2 inhibitor) (25 µm), but [21] not CAY (lipase activity inhibitor) (50 nM) [22]. However, PEDF had no impact on the level of ASC protein under hypoxic conditions (Figure 3B,C). Real-time RT-PCR revealed that the expression of IL-1β/18 mRNA was significantly increased in cardiomyocytes undergoing hypoxia. However, compared with the control group, treatment with PEDF and Z-YVAD-FMK had no effect on the mRNA expression of IL-1β/18 (Figure 3D). Western blot analysis showed that the protein levels of pro-IL18/1β and IL-1β/18 were significantly increased in cardiomyocytes. However, the pro-IL18/1β and IL-1β/18 protein levels were significantly lower in the PEDF and Z-YVAD-fluoromethylketone (Z-YVAD-FMK) group compared with the control group (Figure 3E). ELISA results showed that under the condition of hypoxia, the secreted levels of IL-1β and IL-18 in the cultured supernatant of neonatal cardiomyocytes were lower in the PEDF group than the hypoxia group, and the effect of PEDF was abolished by PEDFR-RNAi-LVA (SiPEDFR) and BEL, but not SiLR and CAY (Figure 3F). The TUNEL result also revealed that hypoxia increased cell death with nucleus DNA damage, while PEDF protected neonatal cardiomyocytes against hypoxia-induced apoptosis and necroptosis though PEDFR. BEL and CAY both partly abolish this protective effect of PEDF under the hypoxic condition (Figure 3G). Z-YVAD-FMK (caspase-1 inhibitor) was able to reduce the secreted levels of IL-1β and IL-18 and the cardiomyocytes’ apoptosis (Figure 3F,G). These results suggested that PEDF inhibits the activity of the NLRP3 inflammasome via PEDF-R/iPLA2 and protects against hypoxia-induced apoptosis in neonatal cardiomyocytes.

### 2.4. PEDF Inhibited Hypoxia-Induced Mitochondrial Fission via PEDF-R/iPLA2

In order to evaluate whether PEDF can inhibit hypoxia-induced mitochondrial fission, which leads to mitochondrial dysfunction, the neonatal cardiomyocytes were treated with PEDF under the hypoxic condition; RNA interference assays were used to silence PEDFR, and BEL was used to block the activation of iPLA2.

**Figure 4:**
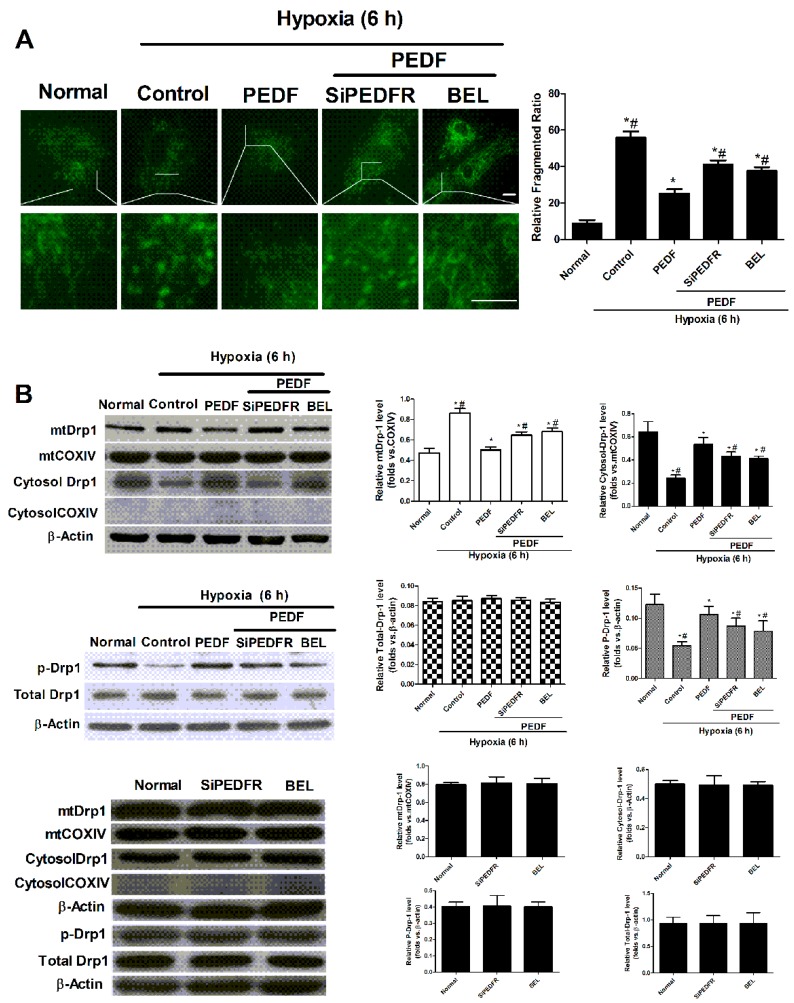
Mitochondrial fission in the neonatal cardiomyocytes. The neonatal cardiomyocytes were exposed to the normoxic or hypoxic condition for 6 h. After hypoxia, cells were treated with PEDF, PEDF + PEDFR siRNAs and PEDF + BEL. (**A**) The structure of the mitochondrial network was analyzed in neonatal cardiomyocytes. The staining of mitochondria was performed with Mitotracker Green. Original magnification 60× (scale bar, 20 μm; *n* = 4. * *p* < 0.05 vs. the control group; # *p* < 0.05 vs. the PEDF group); (**B**) Drp1 protein was assayed by Western blotting in cytosol or mitochondrial membrane (*n* = 4. * *p* < 0.05 vs. the control group; # *p* < 0.05 vs. the PEDF group). Data are expressed as the mean ± SD.

Mito-Tracker^®^ Green showed that PEDF prevents the elongated and interconnected mitochondria from turning into numerous smaller spherical mitochondria; this effect was abolished by SiPEDFR and also reversed by BEL (Figure 4A). Dynamin-related protein 1 (Drp1) is a protein essential for mitochondrial fission. The division of the mitochondria is partly controlled by the phosphorylation of Drp1 at Ser637. Western blotting showed that mitochondrial Drp1 increased during hypoxic conditions, following the decrease of cytosol Drp1. This finding showed that Drp1 translocated from the cytoplasm to the mitochondria under the hypoxic condition. PEDF repressed the Drp1 translocation; SiPEDFR and the iPLA2 inhibitor could partly reverse the effect of PEDF. Western blotting results also showed that the phosphorylation of Drp1 at Ser637 was lower in the hypoxia group than the normal group, while PEDF can increase the phosphorylation of Drp1 at Ser637. Meanwhile, SiPEDFR and the iPLA2 inhibitor could partly reverse this effect of PEDF (Figure 4B). All of these results suggested that PEDF can inhibit hypoxia-induced mitochondrial fission via PEDF-R/iPLA2.

### 2.5. PEDF Inhibited the Hypoxia-Induced Decrease of Mitochondrial Function

Balancing between fission and fusion events is essential for proper mitochondrial function. Recently, extensive research has also revealed that increased mitochondrial fission is a key factor in mitochondrial dysfunction. Excessive mitochondrial fission reduced mitochondrial membrane potential and increased the release of mitochondrial contents.

**Figure 5:**
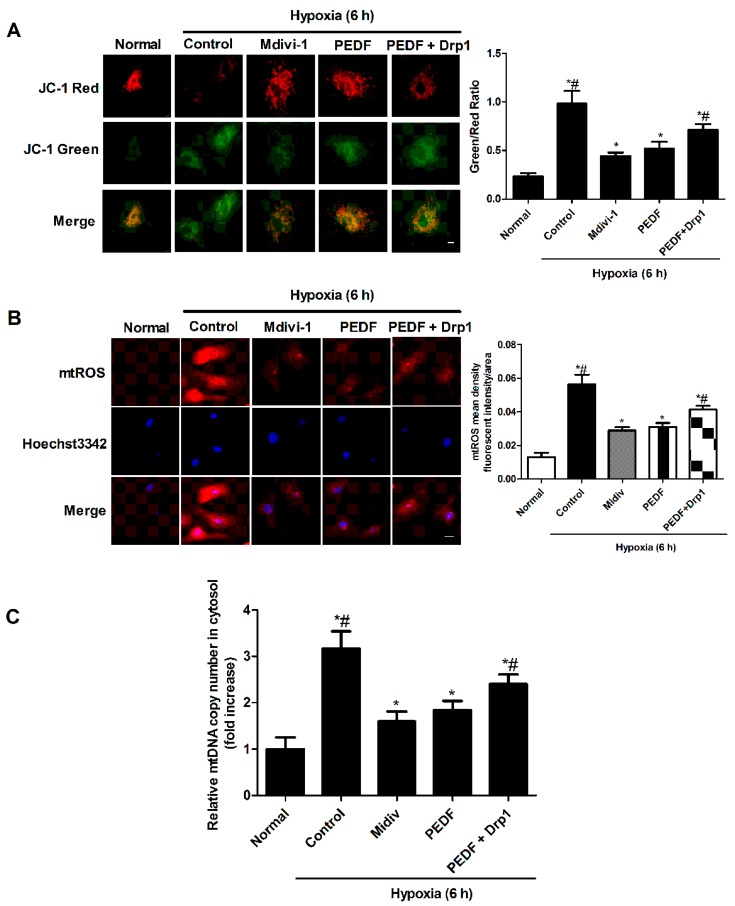
The change of mitochondrial function and mtDNA and mtROS release into cytosol. The neonatal cardiomyocytes were exposed to the normoxic or hypoxic condition for 6 h. Mdivi-1 (1 µm) was used to block the mitochondrial fission; Drp1 plasmids were used to overexpress Drp1 protein. (**A**) JC-1 analysis of the mitochondrial membrane potential in the neonatal cardiomyocytes. Original magnification 60× (scale bar, 20 μm; *n* = 6. * *p* < 0.05 vs. the control group; # *p* < 0.05 vs. the PEDF group); (**B**) mtROS production was monitored by MitoSOX™ Red in neonatal cardiomyocytes. ROS production was observed by red fluorescence of MitoSOX™ by fluorescence microscopy. Original magnification 40× (scale bar, 20 μm; *n* = 6 * *p* < 0.05 vs. the normal group; # *p* < 0.05 vs. the PEDF group); (**C**) the cytosolic mtDNA copy number was measured by quantitative PCR (*n* = 4. * *p* < 0.05 vs. the normal group; # *p* < 0.05 vs. the PEDF group). Data are expressed as the mean ± SD.

The effect of PEDF on mitochondrial membrane potential was evaluated with 5,5’,6,6’-Tetrachloro-1,1’,3,3’-tetraethylbenzimidazolylcarbocyanine iodide (JC-1) staining. The green to red ratio of treated cells markedly increased relative to the control cells, indicating the loss of ∆Ψ. Our results suggested that hypoxia induced the depolarization of the mitochondrial membrane potential, and this disadvantage was abolished by Mdivi-1. PEDF successfully attenuated the hypoxia-induced ∆Ψ depletion; this effect was partly reversed by the overexpression of Drp1 (Figure 5A). The effect of PEDF upon the release of mitochondrial contents was examined with mtDNA and mtROS, which serve as activators of the NLRP3 inflammasome. Hypoxia induced cytosolic accumulation of mtDNA and mtROS in neonatal cardiomyocytes. Mdivi-1 could decrease the release of mtDNA and mtROS into cytosol. Moreover, we also found that PEDF treatment also greatly decreased the copy number of cytosolic mtDNA and mtROS in hypoxia-treated cells. Additionally, PEDF’s effect was partly abolished by the overexpression of Drp1 (Figure 5B,C). These results suggested that PEDF can protect against the hypoxia-induced decrease of mitochondrial function through inhibiting mitochondrial fission, such as depolarization of the mitochondrial membrane potential and mtDNA and mtROS release into cytosol via preventing mitochondrial function.

### 2.6. PEDF Inhibited Mitochondrial Fission-Induced NLRP3 Inflammasome Activation in Neonatal Cardiomyocytes under the Hypoxic Condition

**Figure 6:**
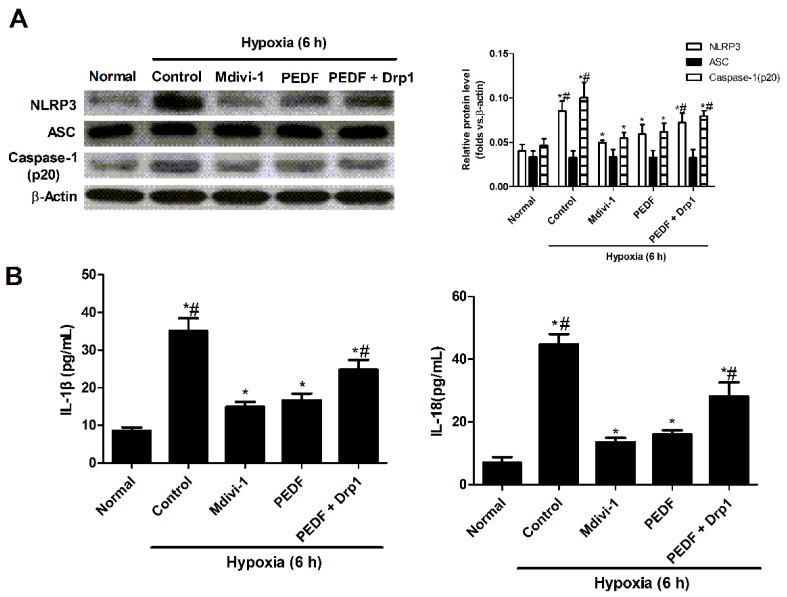
The expression of the NLRP3 inflammasome in neonatal cardiomyocytes. Cells were exposed to the normoxic or hypoxic condition for 6 h. Mdivi-1 (1µm) was used to block the mitochondrial fission under the hypoxic condition. Drp1 plasmids were used to overexpress Drp1 protein. (**A**) Western blot analyzed the expression of NLRP3, ASC and caspase-1 (p20) (*n* = 4; * *p* < 0.05 vs. the normal group; # *p* < 0.05 vs. the control group); (**B**) the protein levels of IL-1β and IL-18 were analyzed in the cultured supernatants of neonatal cardiomyocytes (*n* = 4; * *p* < 0.05 vs. the normal group; # *p* < 0.05 vs. the PEDF group). Data are expressed as the mean ± SD.

To further verify that PEDF inhibited NLRP3 inflammasome activation was regulated by mitochondrial fission, Drp1 plasmids were used to overexpress Drp1 protein, and we analyzed the expression of NLRP3 inflammasome and assessed IL-1β and IL-18 secretion in response to NLRP3 inflammasome triggers. Western blot showed that PEDF decreased the level of NLRP3 inflammasome (NLRP3, caspase-1 (p20)) compared to the hypoxia group (Figure 6A). Overexpression of Drp1 could partly reverse PEDF’s effect. IL-1β/IL-18 had the same tendency (Figure 6B). These results suggested that PEDF inhibited NLRP3 inflammasome activation though preventing mitochondrial fission in neonatal cardiomyocytes under the hypoxic condition.

## 3. Discussion

**Figure 7:**
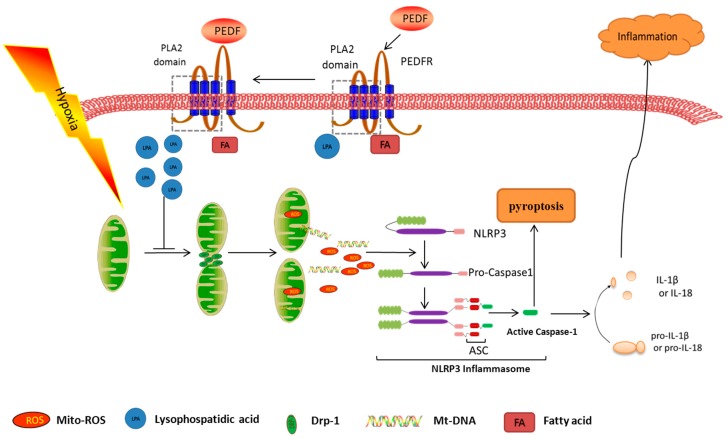
Mechanisms for hypoxia-mediated expression of the NLRP3 inflammasome and the protective effects of PEDF. Hypoxia activates the NLRP3 inflammasome in cardiomyocytes, resulting in caspase-1 activation, and increases IL-1β, IL-18 maturation and release. PEDF binds to PEDF-R to stimulate iPLA2 activity, which is essential for PEDF inhibiting the activation of the NLRP3 inflammasome. The possible protective effects of PEDF may be relevant to the inhibition of mitochondrial fission, thus blocking mtDNA and mtROS release into cytosol and then inhibiting the activation of NLRP3. (The arrows indicate promotion and T-bar indicates inhibition.)

In this study, we found for the first time that PEDF inhibits the activation of the NLRP3 inflammasome in hypoxia cardiomyocytes through PEDFR/iPLA2. The possible protective effects of PEDF may be relevant to the inhibition of mitochondrial fission (Figure 7). These findings suggested that PEDF might be a novel therapeutic strategy for cardioprotection though inhibiting the activation of the NLRP3 inflammasome during an episode of lethal myocardial ischemia injury.

Inflammasomes are multiprotein complexes, which are located in the cytoplasm of the cell and promote the maturation of proinflammatory cytokines (interleukin-1β (IL-1β) and IL-18), as well as highly inflammatory forms of cell death, such pyroptosis [23]. The NLRP3 inflammasome exists in the pathogenesis of a variety of diseases, including genetically-inherited autoimmune conditions and many chronic diseases [9]. An accumulating body of evidence has also indicated that the NLRP3 inflammasome plays an important role in the development of myocardial I/R injury [24]. Sandangeretal et al. reported that the NLRP3 inflammasome was predominantly upregulated in the left ventricle after myocardial infarction (MI) and primarily located in cardiac fibroblasts, which contributes not only to the myocardial fibrosis process, but also cardiac disease development due to the production of autocrine/paracrine factors [5]. Eleonora Mezzaroma and her colleagues described active NLRP3 inflammasomes present in cardiomyocytes bordering the infarct zone later during the infarct process, and they also observed that the induction of the inflammasome led to a significant increase in caspase-1 activity accompanied by a dose-dependent increase in cell death. Our study is consistent with these findings to show that the inflammasome was present in cardiomyocytes 7 days after AMI. In the current study, we also used cultured neonatal cardiomyocytes to show that the inflammasome was present in the hypoxic cardiomyocytes and ultimately induced cardiac cell death.

PEDFR and the laminin receptor (LR) are two putative receptors for PEDF [17]. We suspected that distinct PEDF receptors elicit divergent signals to cause different biological effects [25]. Takanori Matsui et al.’s observations suggest that PEDF could have anti-angiogenic, anti-inflammatory and anti-thrombogenic properties in cultured myeloma cells through the interaction with LR, but not PEDFR [17]. However, in our study, we found that PEDF inhibited hypoxia-induced NLRP3 inflammasome activation via PEDFR, not LR, in cardiomyocytes, which shows a new direction for the treatment of ischemic heart disease though inhibiting the innate immune response. Most of all, PEDF binds to PEDFR and activates the enzyme activity of the enzymatic phospholipase A2 and triglyceride lipase [20]. Our previous study has declared that PEDF and the PEDF-derived peptide 44 mer stimulate cardiac triglyceride degradation via ATGL lipolysis activity to improve cardiac function after infarction [26]. Our group found that PEDF and the PEDF-derived peptide 44 mer inhibit oxygen-glucose deprivation-induced oxidative stress through PEDFR/iPLA2 [27]. However, Preeti Subramanian and his colleagues reported that pigment PEDF prevents retinal cell death via PEDF-R and PLA2 enzymatic activity, and it is critical for upregulating Bcl-2 and cell survival [28]. We are not clear which activity played a role to inhibit the activation of the NLRP3 inflammasome. In this study, we found that PEDF could inhibit the activation of the NLRP3 inflammasome in hypoxia cardiomyocytes through PEDFR/iPLA2.

Our study suggests that excessive mitochondrial fission occurs in the heart in response to AMI and hypoxia cardiomyocytes in vitro; PEDF, on the other hand, could inhibit excessive mitochondrial fission though PEDFR/iPLA2. Mdivi-1 (mitochondrial fission inhibitor) inhibits the activation of the NLRP3 inflammasome in neonatal cardiomyocytes. Thus, we hypothesized mitochondrial fission is involved in the process of PEDF mediating the decrease of the NLRP3 inflammasome. Mitochondria are dynamic organelles that constantly perform fission and fusion. These processes are important for the maintenance of mitochondrial functions [29,30]. Mitochondrial fragmentation is generally referred to as a disease state. Impaired fission directly impacts mitochondrial function, for example resulting in excessive production of free radicals, altered mitochondrial enzymatic activities, impaired calcium homeostasis, low ATP production and overall reduced energy metabolism in mammalian cells [31]. In this study, we found that hypoxia induced excessive mitochondrial fission, which produced high levels of mtROS and mtDNA in the cytosol that exhibit defective mitochondrial function. Previous studies have indicated that mtROS and mtDNA might play an important role in NLRP3 activation [32,33]. Thus, we concluded that hypoxia induced excessive mitochondrial fission, which produced high levels of mtROS and mtDNA in the cytosol-activated NLRP3 inflammasome.

In this study, our results showed that PEDF can inhibit Drp1-mediated mitochondrial fission in cardiomyocytes during hypoxia, which was abrogated by Drp1 overexpression. Drp1 is a member of the dynamin superfamily of GTPases. Drp1, which is mainly cytosolic, translocates to scission sites on the outer mitochondrial membrane to induce the fission or division of mitochondria [30]. In vivo pretreatment of the adult murine heart with a single intravenous bolus of the Drp1 inhibitor reduced MI size and limited the extent of mitochondrial fragmentation [34]. Mitochondrial fission is inhibited when DRP1 is phosphorylated at Ser637 [35]. DRP1 dephosphorylated at Ser637 by calcineurin is recruited to the mitochondrial outer membrane and induces mitochondrial fission [36]. Our results suggested that the PEDF could prevent Drp1 from translocating to the mitochondrial membrane and the dephosphorylation of DRP1 at Ser637 via PEDFR/iPLA2. Therefore, we surmised that the PEDFR/iPLA2 activity would lead to the release of fatty acids and lysophospholipids from phospholipid substrates present in the lipid bilayer of plasma membranes. The products activate relevant signaling pathways to regulate DRP1-induced mitochondrial fission.

In conclusion, our study indicated that PEDF can protect against the hypoxia-induced activation of the NLRP3 inflammasome by inhibiting mitochondrial fission though PEDFR/iPLA2 in cardiomyocytes. Such properties of PEDF could make it a strong candidate for studying ischemic heart disease.

## 4. Experimental Section

### 4.1. Reagents

Anti-NLRP3 (#19771-1-AP) antibody was purchased from Proteintech, Inc. (Proteintech, Rosemont, IL, USA). Anti-ASC antibody (#ST1121)/anti-caspase-1 (p20) antibody (#AP1043) were purchased from CHEMICON (CHEMICON, USA). Monoclonal anti-actin (α-sarcomeric) antibodies (#A2172) were purchased from Sigma-Aldrich Co (St. Louis, MO, USA). Anti-PEDF (sc-16956)/anti-PEDFR (sc-50220)/anti-LR (sc-573) antibody were purchased from Santa Cruz Biotechnology, Inc. (Santa Cruz, CA, USA). Anti-Drp-1 (#8570)/p-Drp-1 (#4867) antibody were purchased from Cell Signaling Technology, Inc. (Cell Signaling, MA, USA). Anti-β-actin (#13E5) antibody was purchased from Cell Signaling Technology, Inc. (Cell Signaling, MA, USA). pro-IL-18, IL-18, pro-IL-1b and IL-1b were from R and D Systems, Minneapolis, MN, USA. Z-YVAD-FMK (caspase-1 inhibitor) (#1141-5) was from BioVision Incorporated, Milpitas, CA, USA. The MitoSOX™ Red mitochondrial superoxide indicator was purchased from Life Technologies Corporation (LIFE, Waltham, MA, USA). Mito-Tracker^®^ Green was purchased from Invitrogen, Inc. (Paisley, UK). The Hoechst 33342/JC-1Apoptosis Detection Kit (JC-1) was purchased from Keygen Biotech, Co. (Nanjing, China). ELISA 1β/18 was purchased from Uscn Life Science Inc. (Wuhan, China). DNeasy Blood & Tissue Kit (50) (#69504) was purchased from QIAGEN GmbH, Co. (QIAGEN, Hilden, Germany).

### 4.2. Recombinant Lentivirus Constructs and Viral Production

Recombinant PEDF-lentivirus (PEDF-LV) was prepared as described previously [15]. PEDF overexpression plasmids and the RNAi vector were packaged in 293T cells. PEDF-R-RNAi-LVA and LR-RNAi-LV of the PEDF-R and LR gene producing PEDF-R shRNA were also successfully constructed, and the concentrated titer of the virus suspension was 2 × 10^12^ TU/L.

### 4.3. Preparations of PEDF Protein

Recombinant rat PEDF (GenBank™ Accession Number NM_177927) was synthesized by Cusabio Biotech, Co., Ltd. (Wuhan, China).

### 4.4. Animal Model and Intramyocardial Gene Delivery

Myocardial ischemia was induced by ligation of the left-anterior descending coronary artery (LAD) in anesthetized rats, as described previously [37]. Sprague-Dawley male rats (8–10 weeks old, weighing 210–250 g) were anesthetized and artificially ventilated. With the animal lying flat, left thoracotomy was performed through the fourth intercostal space, and the LAD was ligated with 6-0 silk suture (Ethicon, Johnson & Johnson, Somerville, NJ, USA) under direct vision. For intramyocardial gene delivery, PEDF-LV in 20 μL enhanced infection solution (ENIS, pH 7.4) was delivered with a 20-μL syringe and 25-gauge needle into the myocardium along the infarct border immediately after surgery. Control animals received an equivalent volume of LV vector in ENIS. The chest cavity was then closed, and animals were extubated and allowed to recover. The animal models were randomly divided into three groups: Group A (normal); Group B (AMI), the animal did not undergo any gene transfer after surgery; Group C (AMI + PEDF), PEDF-LV was transferred after surgery; the animals were sacrificed with an overdose of sodium pentobarbitone (60 mg/kg, i.v.), and their hearts were harvested at the end of 7 days after AMI for further analysis. All of the experiments conform to the Guide for the Care and Use of Laboratory Animals published by the U.S. National Institutes of Health (NIH Publication, 8th Edition, 26 September 2011). The animal care and experimental protocols were approved by the Xuzhou Medical College Committee on Animal Care.

### 4.5. Neonatal Cardiomyocytes Isolation, Culture and Transfection

Cardiomyocytes were isolated from 1-day-old new-born Sprague-Dawley rats [38]. Briefly, neonatal rats were sacrificed by rapid decapitation, and hearts were rapidly removed and placed into dishes on ice; then, hearts were dissected and minced into 1-mm^3^ pieces with sharp scissors, then transferred to a sterile tube. The minced tissue was digested in a PBS solution supplemented with 0.5% trypsin, 0.1% collagenase and 0.02% glucose for 5 min at 37 °C. Then, cells were incubated for 1 h in the presence of 0.1 mmol/L bromodeoxyuridine to selectively enrich for cardiomyocytes. The inclusion of BRDU resulted in the inhibition of the growth of cardiac fibroblasts. The cells were trypsinized and plated (10,000–12,000 cells/cm^2^) in 60-mm dishes for the NLRP3/ASC/caspase-1 (p20) and PEDF/PEDFR/LR assay or in a 48-well culture plate (Corning, New York, NY, USA) for hypoxia treatments and immunofluorescence staining in DMEM/low glucose (Hyclone) supplemented with 10% fetal bovine serum and 100 mg/mL penicillin/streptomycin at 37 °C in a humidified atmosphere containing 5% CO_2_. The resultant cell suspension (10,000–12,000 cells/cm^2^) was plated onto a 48 well culture plate. Hypoxia was achieved by culturing the cells in D-Hank’s liquid with glucose deprivation in a tri-gas incubator (Heal Force, Shanghai, China) saturated with 5% CO_2_/1% O_2_ at 37 °C for the indicated times. For overexpression of Drp1, cells were transfected with the indicated plasmids (pcDNA3.1-Drp1-HA) using Lipofectamine 2000 (Invitrogen) according to the manufacturer’s instructions.

### 4.6. Measurement of mtDNA in Cytosol by Quantitative Real-Time PCR

The DNeasy Blood & Tissue Kit (QIAGEN) kit was used to extract the total DNA of the cardiomyocytes [39]. The mtDNA in cytosol was extracted further by using a multiple centrifugation method as described in Kiichi Nakahira’s article [33]. The mtDNA copy number was measured by real-time polymerase chain reaction (PCR) using the SYBR Green PCR Master Mix (Applied Biosystems, Waltham, MA, USA). In order to determine the relative mitochondrial DNA level, the mtDNA copy number was normalized to nuclear DNA levels in a ratio of cytochrome c oxidase 1 (mtCOI) DNA over nuclear DNA (encoding 18S ribosomal RNA) [40,41]. The following primers were used: 18S forward, 5′-GACTCAACACGGGAAACCTC-3′; 18S reverse, 5′-AGACAAATCGCTCCACCAAC-3′; and rat cytochrome coxidase 1 (COI) forward, 5′-CAGCCGTCCTACTACTTCTCTCA-3′; rat COI reverse, 5′-GATTGGGTCTCCACCTCCA-3′.

### 4.7. Quantitative qPCR-Based Gene Expression

TRIzol (Invitrogen, Carlsbad, CA, USA) was used to extract the total RNA from cells following stimulation experiments. Real-time quantitative PCR was performed on a Light Cycler 480II (Roche, Basel, Switzerland) using SYBR Green PCR Master Mix (Applied Biosystems). The results were normalized to the expression of the β-actin gene. The sequences of the forward and reverse primer were 5′-TAAAGACCTCTATGCCAACACAGT-3′ and 5′-CACGATGGAGGGCCGGACTCATC-3′ for the β-actin gene, 5′-GCACAGTTCCCCAACTGGTA-3′ and 5′-TGTCCCGACCTTGCTGTTT-3′ for the IL-1β gene and 5′-ACCGCAGTAATACGGAGCAT-3′ and 5′-TCTGGGATTCGTTGGCTGTT-3′ for the IL-18 gene.

### 4.8. Detection of Mitochondrial ROS Production

The rat neonatal cardiomyocytes were seeded into each well of a 48-well plate. After cultivation for 5 days, cells were stimulated for respective treatments and subsequently loaded with 200 μL MitoSOX™ (Invitrogen) (5 mM stock in ethanol dissolved in HBSS to a working solution of 5 μM) for 10 min. After three washing steps with PBS, nuclei were counterstained with Hoechst 3342 stain for 15 min. The sample was observed by a fluorescence microscope system (Olympus IX73, Tokyo, Japan).

### 4.9. Staining of Cells with a Mitochondrion-Selective Dye, Mito-Tracker^®^ Green

The rat neonatal cardiomyocytes were seeded into each well of a 48-well plate. After treatment, Mito-Tracker^®^ Green (Molecular Probes, Paisley, UK) (0.5 μm) was added for an additional 30 min at 37 °C. Subsequently, the cells were washed with phosphate buffer saline (PBS) (10 mM Na/K-phosphate buffer (pH 7.0), 0.9% (wt/vol) NaCl), as previously described. The stained cells were observed under a fluorescence microscope (Olympus IX73, Tokyo, Japan).

### 4.10. Measurement of Mitochondrial Membrane Potential

Mitochondrial membrane potential (MMP) was evaluated by the cationic dye JC-1. In normal cells, JC-1 aggregates in mitochondria, fluorescing red. In apoptotic cells, JC-1 accrues in the cytosol, as a green fluorescing monomer. Cells were seeded into each well of a 48-well plate. After PBS washing three times, cells were incubated with JC-1 10 μg/mL for 15 min at 37 °C in the dark. Cells were washed, suspended in PBS and analyzed by fluorescence microscope (Olympus IX73, Tokyo, Japan).

### 4.11. Western Blotting Analysis

For Western blotting analysis, the cells were solubilized in lysis buffer (100 mmol/L Tris-HCl, 2% SDS, 10% glycerin, 100 mmol/L dl-Dithiothreitol (DTT) and protease inhibitors, pH 6.8). Protein extraction of both the cytosolic and mitochondrial fractions was performed using a multiple centrifugation method as described previously [42]. Proteins were separated on 10% polyacrylamide gels and then electrotransferred onto the nitrocellulose membrane (Millipore, Billerica, MA, USA). After blocking for 3 h with 3% bovine serum albumin (BSA) in Tris-buffered saline with 0.1% Tween-20 (TBST), membranes were incubated overnight at 4 °C with primary antibodies in TBST containing 3% BSA and then fluorescently labeled secondary antibody (Rockland, Limerick, PA, USA) for 1 h at room temperature. After washing, the protein bands were scanned by the Odyssey Infrared Imaging System (Li-Cor Biosciences, Waltham, MA, USA).

### 4.12. Immunofluorescence

Cells were grown in 48-well plates. After the respective treatments, cells were washed twice with PBS (phosphate-buffered saline) and fixed with freshly-prepared 4% paraformaldehyde at room temperature for 15 min. For heart tissue staining, frozen myocardial tissue was horizontally sliced into a 5-μm section and mounted on a glass slide. Antigen accessibility was increased by treatment with 2% Triton X-100 for 10 min. Then, cells were blocked with 3% BSA for 30 min. Following incubation with neonatal and secondary antibodies, cells were incubated with neonatal antibodies overnight at 4 °C. After washing three times with PBS, cells were stained with a secondary antibody for 1 h at room temperature. The nuclei were counterstained with DAPI for 15 min. Each time after the operation, cells were washed thrice with PBS and each time for 5 min. Cells were captured and analyzed using TCS SP8 STED 3X (Leica, Germany).

### 4.13. Transmission Electron Microscopy

Samples of heart tissue were fixed with 2.5% glutaraldehyde overnight. Subsequently, samples were incubated while protected from light with 1% osmium tetroxide for 2 h. After washing in distilled water, the samples were incubated in 2% uranyl acetate for 2 h at room temperature and then dehydrated in graded ethanol concentrations. Finally, the samples were embedded in molds with fresh resin. Ultrathin sections were obtained with an EM UC7 (Leica, Solms, Germany), stained with lead citrate and examined with a Tecnai G2 T12 (FEI, Hillsboro, OR, USA).

### 4.14. Enzyme-Linked Immunosorbent Assay

IL-1β and IL-18 in cultured cardiomyocyte supernatants were measured using commercial ELISA kits (Uscn Life Science, Wuhan, China).

### 4.15. Statistical Analysis

Data are expressed as the mean ± SEM. Statistical analysis of the results was carried out using one-way analysis of variance (ANOVA) followed by the Tukey–Kramer post-hoc test and independent samples *t*-test. Analysis involved use of SPSS v18.0 (SPSS, Chicago, IL, USA). *p* < 0.05 was considered to be statistically significant.

## 5. Conclusions

In conclusion, this study extended a new and beneficial role for PEDF on inhibiting the activation of the NLRP3 inflammasome and revealed the molecular mechanism by which PEDF mediates this effect. We first reported that PEDF inhibits the activation of the NLRP3 inflammasome both in vivo and in vitro. Meanwhile, recent research has found that endogenous cytosolic mtDNA and mtROS can serve as activators of NLRP3 inflammasome activity. In this study, PEDF reduced mitochondrial fission and mitochondrial fission-induced mitochondrial DNA (mtDNA) and mitochondrial ROS (mtROS) release into cytosol through PEDFR/iPLA2. In addition, we also found that Mdivi-1 (mitochondrial fission inhibitor) inhibited the hypoxia-induced activation of the NLRP3 inflammasome and the release of mtDNA and mtROS. These findings suggested that PEDF might be a novel therapeutic strategy for cardioprotection though inhibiting the activation of the NLRP3 inflammasome during an episode of lethal myocardial ischemia injury.

## Supplementary Materials

Supplementary materials can be found at www.mdpi.com/1422-0067/17/12/2064/s1.
