# Supplementary material for: PEDF Inhibits the Activation of NLRP3 Inflammasome in Hypoxia Cardiomyocytes through PEDF Receptor/Phospholipase A2
Source: Int J Mol Sci. 2016 Dec 12;17(12):2064. doi: 10.3390/ijms17122064 (PMC5187864; doi:10.3390/ijms17122064)
Supplement: Supplementary file 1 [file ijms-17-02064-s001.pdf]

Supplementary Materials: PEDF Inhibits the Activation of NLRP3 Inflammasome in Hypoxia Cardiomyocytes through PEDF Receptor/Phospholipase A2

Zhongxin Zhou, Zhu Wang, Qiuhua Guan, Fan Qiu, Yufeng Li, Zhiwei Liu, Hao Zhang, Hongyan Dong and Zhongming Zhang

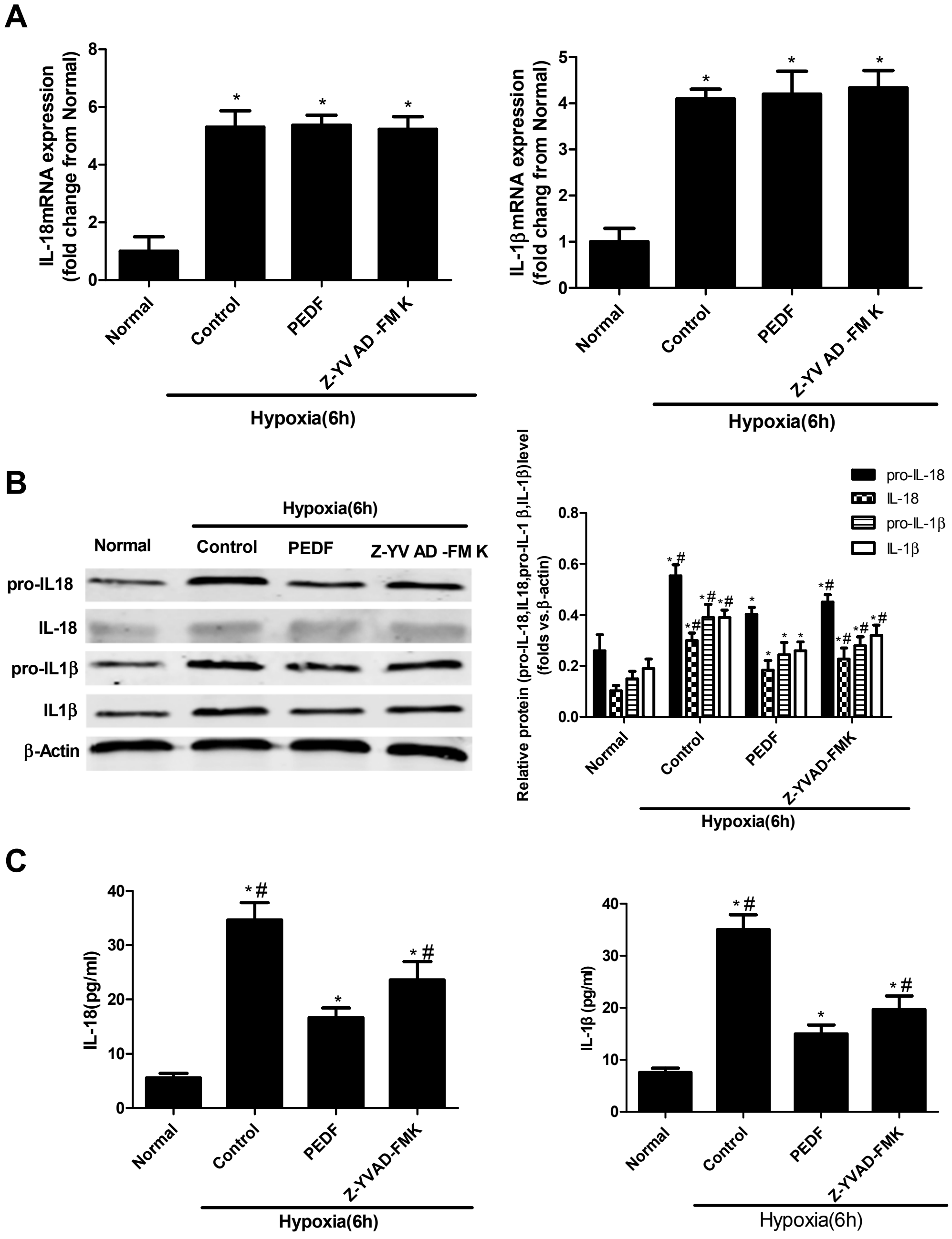

Figure S1. Effects of PEDF on the mRNA expression of IL-1β/18 and the protein levels of pro-IL18/1β and IL-1β/18. (**A**) IL-1β/18 mRNA expression was examined by real-time PCR. The results were expressed as the relative expression to β-actin and plotted as the ratio of the control group ($n = 4$; * $p < 0.05$ vs. the control group); (**B**) Representative Western blot analyses of pro-IL18/1β and IL-1β/18 expression ($n = 4$; * $p < 0.05$ vs. the control group; # $p < 0.05$ vs. the PEDF group); (**C**) ELISA analyzed the concentrations of IL-1β/18 in the culture medium ($n = 4$; * $p < 0.05$ vs. the control group; # $p < 0.05$ vs. the PEDF group). Data are expressed as the mean ± SD.

PEDF inhibited the hypoxia-induced increase of pro-IL18/1β and IL-1β/18 protein levels and secreted levels Real-time RT-PCR revealed that the expression of IL-1β/18 mRNA was significantly increased in cardiomyocytes undergoing hypoxia. However, compared with the control group, treatment with PEDF and Z-YVAD-FMK had no effect on the mRNA expression of IL-1β/18 (Figure S1A). Western blot analysis showed that protein levels of pro-IL18/1β and IL-1β/18 were significantly increased in cardiomyocytes. However, pro-IL18/1β and IL-1β/18 protein levels were significantly lower in the PEDF and Z-YVAD-FMK group compared with the control group (Figure S1B). Similar to the Western blot result, the secreted levels of IL-1β and IL-18 in the cultured supernatant of neonatal cardiomyocytes were significantly lower in the PEDF and Z-YVAD-FMK group compared with the control group (Figure S1C).
